# Editorial: Autism Spectrum Disorders: Developmental Trajectories, Neurobiological Basis, Treatment Update, Volume 2

**DOI:** 10.3389/fpsyt.2020.00589

**Published:** 2020-06-24

**Authors:** Roberto Canitano, Yuri Bozzi, Dirk Dhossche

**Affiliations:** ^1^Child Neuropsychiatry Unit, Siena University Hospital, Siena, Italy; ^2^Center for Mind/Brain Sciences (CIMeC), University of Trento, Trento, Italy; ^3^Department of Psychiatry and Human Behavior, University of Mississippi Medical Center, Jackson, MS, United States

**Keywords:** autism spectrum disorders, neurobiology, developmental trajectories, treatment, children and adolescence

In this collection of articles on Autism Spectrum Disorder (ASD), various themes have been covered with the aim to widen the perspective of updating the readers on the recent advancements in this research field. Continuing expanding areas of investigation have attracted the interest of authors and the resulting Research Topic contains different contributions in different fields of ASD research, including classification, endophenotypes, gender differences, comorbidities, and biological underpinnings.

The publication of DSM-5 and ICD-11 has changed ASD classification and diagnostic criteria (1), introducing ASD as a behaviorally defined neurodevelopmental disorder and eliminating previous diagnoses of Autistic Disorder, Asperger's Disorder, and Pervasive Developmental Disorder—not otherwise specified, present in the previous version of DSM. Oberman and Kaufmann argue in their article that the term Autism Spectrum Disorder, and its criteria, is preferred over the plural form Autism Spectrum Disorders that is still prevalent in the basic science and genetic literature. In addition, they find that the diagnosis of ASD is inaccurate in many individuals with intellectual disability, and advocate to assess and use other diagnostic entities, such as Social Communication Disorder or Stereotypic Movement Disorder, in order to avoid over-diagnosing ASD in those individuals.

Investigating ASD behavioral, functional, and neuroanatomical endophenotypes represents a hot topic in ASD research (2, 3). A number of cognitive models focused on executive function (EF) have been proposed to explain the symptom clusters observed in ASD, as detailed by Demetriou et al. Empirical studies pointed out a broad impairment in EF. The observed heterogeneity of EF performance is considered a limiting factor in establishing EF as a cognitive endophenotype in ASD. Further understanding of the neurobiological basis that underpins EF performance, such as the excitation/inhibition hypothesis, will likely be important to shed light on these components. Importantly, the authors state that application of the research domain criteria (RDoC) framework could improve our understanding of EF impairment in ASD and facilitate targeted interventions. To investigate alterations in neural processing of social visual information in children with ASD, Jan et al. used high density electroencephalography and high-resolution eye-tracking. The study highlighted differences in the neural processing of dynamic cartoons containing human-like social interactions. ASD children, as compared with controls, showed decreased prefrontal and cingulate activation, impaired activation of the premotor cortex, and increased activation of parietal, temporal, occipital, and cerebellar regions. Thus, impairments in brain regions involved in processing social cues are present from an early age in ASD children and deserve further investigation. Spera et al. used machine learning to evaluate altered functional connectivity in resting-state fMRI datasets of individuals with ASD and matched controls. Theirs results indicate that both under- and over-functional connectivity occurred in a selected cohort of ASD children as compared with controls, and that these functional alterations are spread in different brain networks including the precuneus, the inferior frontal gyrus, and the hippocampus. Repetitive transcranial magnetic stimulation (rTMS) is a novel treatment that has been used in a limited number of studies in children with ASD. Yang et al. evaluate the use of high-frequency rTMS over the left inferior parietal lobule in 11 low-functioning children with ASD. This preliminary study provides positive evidence for efficacy and that larger and controlled studies are warranted. In the study by Malatesta et al., the absence of left-cradling shown in mothers of typically developing children was not observed in mothers of ASD children, who exhibited a significant left-cradling bias in the 6–12 months age group. It remains to be further investigated whether this pattern is related to the overstimulation in which ASD mothers try to engage the infants in response to their lack of social interaction. Ruta et al. instead validated the Quantitative Checklist for Autism in Toddlers (Q-CHAT) in an Italian cohort of young children with ASD and developmental disorders, showing that Q-CHAT has good psychometric properties and external validity to distinguish ASD children from both typically-developing children and children with developmental delay.

The significant gender bias in ASD incidence (4:1 male to female ratio) has been postulated to have neurodevelopmental origins (4, 5). However, existing studies indicate minimal sex differences in core ASD symptoms (6). Mahendiran et al. investigated sex differences in social and communication skills in ASD, ADHD, and typically developing children. The authors found that females with ASD had worse performances than males at older ages, in spite of better communication skills in earlier age. This suggests that a developmental approach to find out sex differences over time might have multiple implications. In another study (Mahendiran et al.), the same authors performed a metanalysis of 11 original studies on sex differences in children with ASD and ADHD, and did not detect sex differences in social and communication function. However, the authors found a remarkable heterogeneity between the analyzed studies with respect to psychometric measurements and population differences. In particular, several of the studies included a low number of females, thus likely being underpowered to detect sex differences. Future larger studies, controlling for measure and with adequate numbers of female participants are required to further understand sex differences in social and communication domains.

ASD presents a wide range of comorbidities (7). Scandurra et al. instead evaluated adaptive skills in children with ASD, ADHD, or ASD+ADHD. A worse general adaptive profile was ascertained in the ASD and ASD+ADHD groups, as compared with the ADHD-only group, indicating the load of autism ASD symptoms on overall adaptive profile. The externalizing history of a cohort of young violent offenders with ASD, compared with offenders without ASD, is described in the article by Hofvander et al. A very high prevalence of externalizing and antisocial behaviors in the history of these offenders were detected and there were few differences between the groups. Placements in foster homes were overrepresented in ASD-offenders, which were also overrepresented in sex crimes with a child victim. This portion of ASD individuals causes significant challenges to the criminal justice system and additional knowledge is needed to prevent these individuals from committing crimes and also to receive a fair judicial treatment. Feeding problems are prevalent in children with ASD. In order to examine this further, Catino et al. studied interactions between parents and infants diagnosed with ASD, during feeding, using the Scale for the Assessment of Feeding Interactions (SVIA), a new assessment tool. This study supports the psychometric robustness of the SVIA, highlights the importance of direct observation of the parent-child dyad during feeding, and supports a high rate of feeding problems in children with ASD.

Finally, a group of four papers addressed the biological underpinnings of ASD (8). Oxidative stress and polymorphisms in genes encoding antioxidant enzymes (such as glutathione transferases, GSTs) might be involved in the development of ASD. Mandic–Maravic et al. found specific perinatal complications as significant risk factors for ASD. GSTM1 polymorphism might serve as a moderator of the effect of some prenatal factors on the risk of ASD such as using medication during pregnancy. In their review article, Balasco et al. addressed the neurobiological bases of sensory processing in ASD, with a specific focus of tactile sensitivity. Sensory abnormalities affect 90% of ASD individuals, and are recognized as diagnostic criteria for ASD. The article summarizes the most recent findings in this domain, focusing on both clinical studies and preclinical research on ASD mouse models. Modi et al. described a loss of inhibition that resulted in increased excitation/inhibition balance in the CA2 hippocampal circuit of Neuroligin 3 knockout mouse, a non-syndromic ASD mouse model. These defects were associated to social cognition deficits and confirmed the emerging role of the CA2 hippocampal region in controlling social behaviors. Finally, Sanfeliu et al. used RNA sequencing in *Mecp2* mutant mice and age-matched controls to identify differentially regulated genes and pathways. The authors found that some genes and pathways were differentially expressed in the brain and blood of *Mecp2* mutant mice at a symptomatic, but not pre-symptomatic, stage. Genes controlling circadian rhythms and immune response were specifically enriched in *Mecp2* mutant brain and blood, respectively.

## Author Contributions

All the authors equally contributed to the writing of the manuscript.

## Funding

YB is supported by the University of Trento Strategic Project TRAIN -Trentino Autism Initiative.

## Conflict of Interest

The authors declare that the research was conducted in the absence of any commercial or financial relationships that could be construed as a potential conflict of interest.
